# Isolation and Identification of Pathogenic Bacteria Present in Commercially Important Fish, Shellfish, Water, and Soil Samples of Kaptai Lake, Bangladesh

**DOI:** 10.1111/1758-2229.70252

**Published:** 2025-12-03

**Authors:** Susmita Chakma, Hrishika Barua, Aklima Akter, Shama Afroze, Md. Faisal, Nurul Absar Khan

**Affiliations:** ^1^ Department of Fishing and Post‐Harvest Technology Faculty of Fisheries, Chattogram Veterinary and Animal Sciences University Chattogram Bangladesh; ^2^ One Health Institute, Chattogram Veterinary and Animal Sciences University Chattogram Bangladesh

**Keywords:** bacterial load, bacterial risk assessment, Baghaichari, correlation, pathogenic bacteria, Rangamati

## Abstract

This study aimed to analyse the enteric pathogenic bacteria, in fish, soil, and water collected from Kaptai Lake that could be transmitted to humans through handling and consumption. Various types of selective agar media were used to isolate *Escherichia coli*, *Salmonella*, *Shigella*, *Vibrio cholerae*, *Vibrio parahaemolyticus*, and *Vibrio vulnificus*. After observing the culture morphology, microscopic examination, and biochemical tests were performed. Total plate count indicated varying levels of bacterial load among 20 fish species, *Puntius ticto* exhibiting the highest (8.53 ± 0.21 × 10^6^ CFU/g) microbial load. Among the soil and water samples, Guccha gram (in Baghaichari) exhibited the highest bacterial load (6.13 ± 0.66 × 10^6^ CFU/g and 3.90 ± 0.20 × 10^6^ CFU/mL, respectively). *E. coli* in most water and soil samples suggested potential contamination by human and animal faecal matter. Bacterial risk indices showed that 13 fish species among 20 fish samples were categorised as ‘Good’, indicating safe for consumption, and the remaining 7 fish species were identified as ‘Acceptable’. The correlation matrix showed high correlation scores in most places (from 0.5 to 1), indicating that environmental hygiene matters most for the safety of fish. Higher microbial load in soil, water and fish samples emphasises the necessity for strict adherence to reduce zoonotic diseases.

## 1 Introduction

Kaptai Lake, situated in the Chattogram Hill Tracts of South‐eastern Bangladesh, is renowned not only as the nation's largest manmade lake and a crucial source of hydroelectric power but also as a sanctuary for diverse fish species, contributing significantly to regional biodiversity (Karmakar et al. 2011). With a total average area of 68,800 ha and a water surface area of 58,300 ha this lake set the record to produce 12,695 MT, 13,915 MT, and 20,282 MT of fish in 2019–20, 2020–21, and 2021–22, respectively (Suman et al. 2021; DoF 2023). In order to boost fish production, around 60 MT of fish fry were released into the lake in FY 2021–22 (DoF 2023). The lake has remained a reliable source for fish production, with indigenous major carps such as *Labeo rohita*, *Catla catla*, *Cirrhinus cirrhosus*, *Labeo calbasu* and *Tor tor* dominating the catch, accounting for about 81% immediately after lake formation (Suman et al. 2021). In terms of different aquaculture practices, cage culture, pen culture and creek culture are mostly followed in Kaptai Lake (Giri et al. 2019). However, the capture and culture fisheries in this lake have steadily declined to 5% over the years, while the production of SIS, for instance, *Corica soborna*, *Gudusia chapra*, and so forth, increased to almost 90% of the total catch, which are now two of the most important commercial fish species in Kaptai Lake. Even though fish production in the lake is increasing day by day due to small indigenous species (SIS), larger fishes like IMCs (*Labeo rohita*, *Gibelion catla*, *Cirrhinus cirrhosus*, etc.) are decreasing at an alarming rate. Calculating the standing crops reduces the overall economic return from the catch to 60%, assuming that the Indian major carps (IMCs) volume has been replaced by SIS (Patwary et al. 2016). Reduction in fish biodiversity is attributed to heavy siltation, poor water quality, along with natural and anthropogenic pressures (Patwary et al. 2016; Suman et al. 2021). The Halda River, which runs adjacent to the lake, is renowned as a unique breeding ground for carp in Southeast Asia which has drastically seen a decline, with the loss of 26 fish species due to chronic water pollution stemming from municipal sewage, industrial, and agricultural waste either directly discharged into the river or via seepage and runoff. This pollution has led to frequent eutrophication, excessive turbidity and oxygen depletion in many parts of Kaptai Lake (Ahmed et al. 2010; Suman et al. 2021).

Due to this problem, the microbial safety of the fish species collected from this region remains a matter of great concern. The water in Kaptai Lake has been deemed unsuitable for drinking and domestic use due to the presence of pathogenic microbes such as *Enterococcus* spp., *Salmonella* spp., *Pseudomonas* spp., and *Vibrio* spp., along with elevated levels of toxic metals like lead, cadmium, and nickel (Barua et al. 2016). Moreover, Kaptai Lake provides social, economic, and cultural benefits to the communities. Uncontrolled soil use, dumping of human and animal faecal matter, sewage disposal and manure application cause higher amounts of bacterial load and the presence of pathogenic bacteria in soil samples (Hoque et al. 2021).

Fish can become contaminated by microbes through the earth, air, dust, medical equipment (used in manufacturing or dispensing), humans, or animal secretions (W H Organization 2021). The people residing in the adjacent areas of the lake use the lake water for drinking and other domestic needs without first undergoing any kind of purification treatment (Barua et al. 2016). Analysing the microbiological quality of water and fish is crucial for managing public health and beneficial for maintaining safe export and consumption of fish and fishery products (Faridullah et al. 2016). Wastewater serves as a source of spreading pathogenic bacteria, which can cause waterborne disease outbreaks worldwide (Chen et al. 2017). Environmental water sources are susceptible to contamination by *E. coli* from both humans and other warm‐blooded animals through faecal disposal (Cho et al. 2020). *Salmonella*, one of the most prevalent zoonotic bacteria originating from animals, has recently been recognised as originating from environments as well. *Salmonella‐*contaminated water and crops receiving contaminated irrigation water with *Salmonella* from human and animal sources have been suggested as sources of *Salmonella* infections (Dewey‐Mattia 2018).

In this study, several locations were chosen around Kaptai Lake and Baghaichari upazila based on ecological significance, historic biodiversity data, and the local economic activities of the community to determine bacterial load and the presence of pathogenic bacteria in fish, water, and soil. Adjacent areas of Kaptai Lake, like Reserve Bazar, Bonorupa Bazar, Shuvolong Bazar and so forth, were selected as most of the fish landing activity occurs in these places, as well as having high economic importance. Different places of Baghaichari upazila, like Thana Bazar, Guccha Gram, Bottoli and so forth, were selected as they are the largest upazila (1929 km^2^) in the Rangamati region with a population growth of 2.10% observed in the last century (Hoque et al. 2021). This study evaluated the bacterial load and presence of pathogenic bacteria on fish samples collected from these different areas. In addition, to identify if there is any relationship between the soil and water microbial quality with the fish bacterial load, the bacterial load and presence of those bacteria were also assessed. This study aims to gain insights into how pathogenic bacteria present in sediments and water have the potential to contaminate fish that live near the lake floor.

## 2 Methodology

### 2.1 Sample Collection

#### 2.1.1 Collection of Fish Sample

In this study, a stratified purposeful sampling approach was employed to collect fish samples, with 20 fish samples collected from 20 different locations across two collection sites: the lower region of Kaptai Lake and Baghaichari. Fish species were selected on the basis of availability in the region and 3 fish from each species were collected to reduce error. For example, in the Reserve Market region, the *Sperata aor* fish was more available; in the BFDC landing centre, the *Corica soborna* was more available. A list of which fish were collected from which different areas is given in Table 1. The fish were collected during the early morning hours of the day (between 6:00 am and 8:00 am local time), packed in individual sterilised plastic zip‐locked bags and then kept in the ice boxes to preserve the bacteriological properties of the samples.

**TABLE 1:** List of fish species collected from different areas.

Place of Kaptai Lake	Fish Species	Location	Latitude (°N)	Longitude (°E)
Lower Region of Kaptai Lake	*Corica soborna*	BFDC Landing Centre	22.64834	92.1851
	*Labeo bata*	Bonorupa	22.65772	92.17931
	*Penaeus monodon*	Mosjid Market	22.65279	92.19701
	*Clupisoma garua*	Jalojan Ghat	22.65772	92.17745
	*Glossogobius giuris*	Jholong Ghat	22.393	92.1043
	*Zenarchopterus ectuntio*	Kandemu	22.3915	92.52
	*Sperata aor*	Reserve Bazar	22.65581	92.19589
	*Mastacembelus armatus*	Shuvolong Bazar	22.4121	92.1254
	*Gudusia chapra*	Shuvolong Jhorna	22.4229	92.1433
	*Heteropneustes fossilis*	Shuvolong (Check post)	22.422	92.1558
Baghachari	*Oreochromis niloticus*	Bottoli	23.1543508	92.1914087
	*Neotropius atherinoides*	Beli Bridge	23.1648979	92.18947733
	*Ompok pabda*	Coumohoni	23.1625191	92.190024
	*Puntius ticto*	Guccha Gram	23.1559393	92.1913394
	*Channa marulius*	Marisha Launch Ghat	23.1609697	92.1904537
	*Rohtee cotio*	Paddopara	23.1550821	92.1914308
	*Channa striata*	Puran Bazar	23.1604888	92.1903058
	*Mystus tengara*	Kali Mohon Para	23.1609051	92.1901774
	*Labeo calbasu*	Thana	23.1594527	92.1906376
	*Mystus cavasius*	Thana Bazar	23.155946	92.1913318

#### 2.1.2 Collection of Soil and Water Samples

**FIGURE 1:**
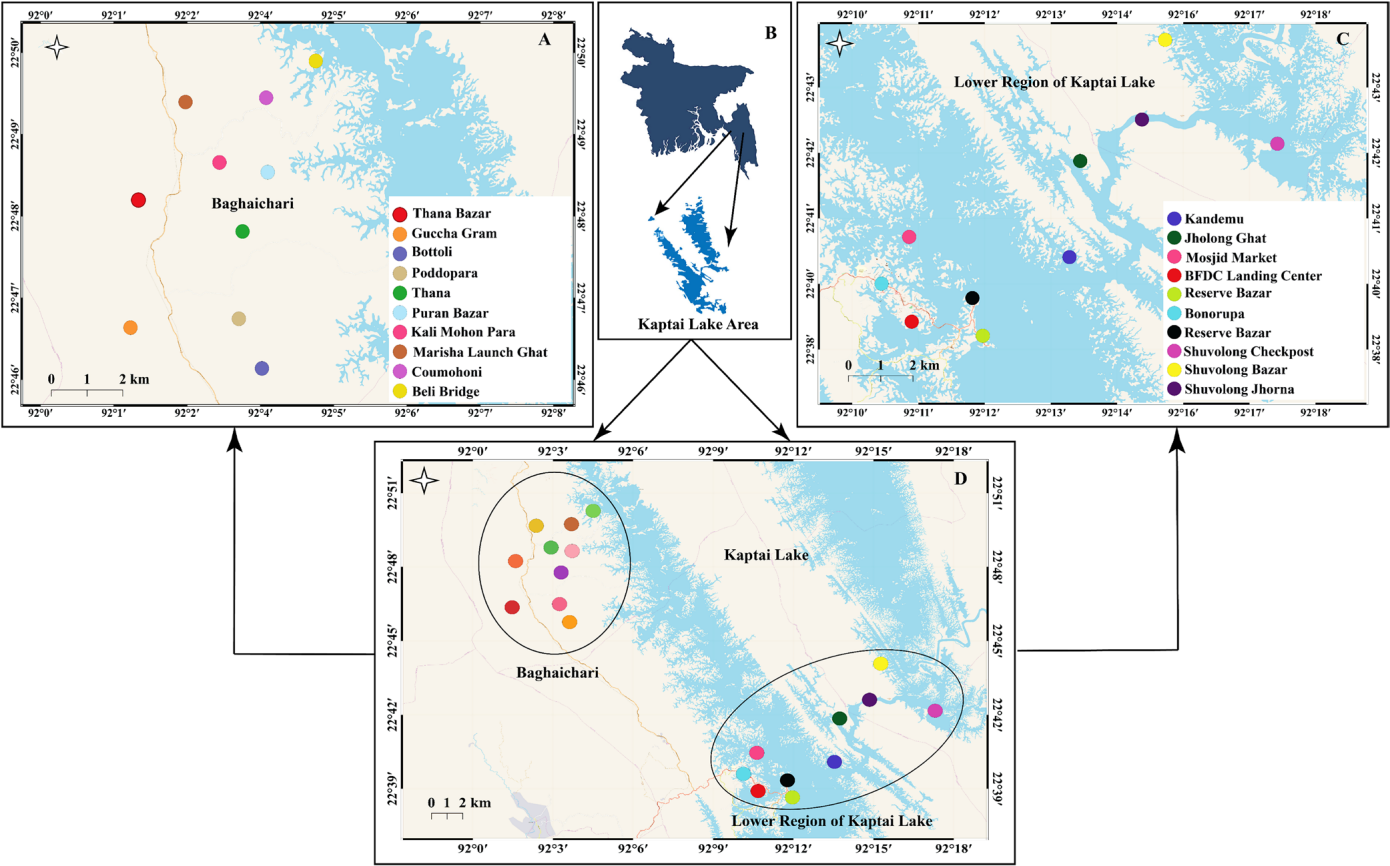
Different sampling sites of Kaptai Lake (A) 10 locations of the Baghachari region; (B) Map of Bangladesh and Kaptai Lake; (C) 10 locations from the lower region of Kaptai Lake; (D) All 20 locations from which samples were collected.

According to FAO and WHO, monitoring aquaculture water bodies like lakes and ponds is important for risk assessment, for which soil and water samples were monitored in this study (Welcomme and Barg 2000). Soil samples were collected from different sampling stations (Figure 1) and packed in sterile zip‐lock poly bags. In addition, from respective sampling stations (Figure 1), water samples were collected in sterile Falcon tubes (50 mL) and placed in sampling buckets containing ice blocks to maintain a temperature of 2°C–8°C. In order to reduce sampling error, three replications per environmental sample (soil and water) were collected. Then all the collected fish, water and soil samples were immediately transported to the Nutrition and Processing Laboratory, Faculty of Fisheries, CVASU for isolation and identification within 5–6 h of sample collection. All samples (fish, soil and water) were collected in replication.

### 2.2 Determination of Total Plate Count

Sample preparation was done using the methodology described by Al Sanjee and Karim (2016). About 10 g of the fish sample was cut from the different parts of the fish body (head, middle, and tail regions) with a sterile knife. The cut samples were transferred into a sterile blender, and 90 mL of sterile peptone water was added to prepare a homogenised sample. For the soil sample, 90 mL of peptone water (HiMedia, Nashik, India) was properly mixed with 10 g of the soil sample. Whereas, for the water samples, 10 mL of water was mixed properly with 90 mL of peptone (Toms et al. 2020). To determine total plate count, the spread plate method was followed using plate count agar (PCA) (HiMedia, Nashik, India) in aseptic conditions. First, 6 dilutions of the samples were prepared in test tubes from 10^−1^ to 10^−6^ by mixing with sterile peptone water in a 1:10 ratio. Using a sterile micropipette, samples from the test tubes with different dilutions were aseptically transferred to these prepared agar plates, lifting the lid just enough to allow the pipette tip's entry. Once the samples were on the plates, they were evenly spread using a sterile, flamed *L*‐shaped glass rod, ensuring the gel was dry. The plates were then placed upside‐down in an incubator at 37°C for 48 h. The dilution and incubation were performed in replication. After 48 h, only plates with 30 to 300 colonies were selected for counting to ensure accuracy using the colony counter.

### 2.3 Isolation and Identification of *E. coli*, *Vibrio Species*, *Salmonella*, and *Shigella*

Pathogenic bacteria, for instance *E. coli*, *Vibrio cholerae*, *V. parahaemolyticus*, *V. vulnificus*, *Salmonella* sp., and *Shigella* sp., were identified according to ISO (International Organisation for Standardisation) standard protocols.

#### 2.3.1 Cultural Examination

##### 2.3.1.1 Detection of *E. coli*

To detect *Escherichia coli* (*E. coli*) in samples, a portion of the sample was first enriched in a selective broth medium, such as MacConkey agar (HiMedia, Nashik, India) and Eosin‐methylene blue (EMB) agar (HiMedia, Nashik, India) and incubated at 37°C for 24 h (Islam et al. 2014).

##### 2.3.1.2 Detection of *V. cholerae*, *V. parahaemolyticus* and *V. vulnificus*

To detect *Vibrio cholerae*, *V. parahaemolyticus*, *and V. vulnificus*, samples were enriched in alkaline peptone water (APW) and incubated at 37°C for 18–24 h. After enrichment, aliquots were streaked onto selective agar media, such as Thiosulfate‐Citrate‐Bile Salts‐Sucrose (TCBS) agar (HiMedia, Nashik, India) and incubated at 37°C for 24 h (Shammi 2015; Yaashikaa et al. 2016).

##### 2.3.1.3 Detection of *Salmonella* and *Shigella* sp.

To detect *Salmonella* and *Shigella* species, samples were first enriched in tetrathionate and selenite broth, followed by incubation at 37°C for 24–48 h. After enrichment, aliquots were streaked onto selective agar media such as Xylose Lysine Deoxycholate (XLD) agar and *Salmonella‐Shigella* (SS) agar (HiMedia, Nashik, India) and incubated at 37°C for 24 h (Shammi 2015; Islam et al. 2014; Aktar et al. 2016).

#### 2.3.2 Microscopic Examination

The isolated bacteria were subjected to microscopic observation, where a single colony from each isolate was placed on a clean slide, heat‐fixed, and subjected to Gram staining. The stained samples were then examined under a light microscope (Model: B‐192 SN 485060, OPTIKA, Italy).

#### 2.3.3 Biochemical Test for Identification

##### 2.3.3.1 Catalase Test

Catalase test was done by mixing a bacterial colony with a few drops of 3% hydrogen peroxide (H_2_O_2_) (Merck Germany) on a slide, and the formation of bubbles was observed within 10 s, indicating a positive result, meaning the presence of catalase enzyme (Reiner 2010).

##### 2.3.3.2 Oxidase Test

In order to perform the oxidase test, a filter paper was soaked with 1% tetramethyl‐p‐phenylenediamine dihydrochloride (HiMedia, Nashik, India), and then the filter paper was dried. Following that, bacterial colonies were rubbed onto the paper, and a colour change was checked within 10 s. A dark purple colour formation indicates a positive result, meaning the presence of cytochrome c oxidase (Winn Washington et al. 2006).

##### 2.3.3.3 Methyl Red (MR)

The isolates were inoculated in Methyl Red—Voges‐Proskauer (MR‐VP) broth (HiMedia, Nashik, India) and incubated at 37°C for 24 h. Then, 5 drops of methyl red (DLC Brand, Bangladesh) were added to the culture as an indicator in the culture tube, and a change of colour was observed. Formation of red colour indicates a positive result and yellow colour means a negative result (McDevitt 2009).

##### 2.3.3.4 Triple Sugar Iron (TSI) Test, Gas and H_2_S Production

To perform the TSI test of isolated bacteria, at first a single colony of the desired bacteria was inoculated into the butt of the TSI agar (HiMedia, Nashik, India) tube. Then, the surface of the slant was streaked, and the cap of the tube was loosely tightened to allow gas exchange. Later, all the tubes were incubated at 37°C for 24 h. The presence of a red or pink slant indicates an alkaline state (K) and a yellow butt represents an acidic state (A). After incubation, the presence of a black precipitate in the slant or butt signifies a positive result, indicating the formation of iron sulfide (FeS) from the reaction between hydrogen sulfide (H_2_S) and iron salts in the agar and the formation of bubbles or cracks in the agar indicates gas production (Harley 2008).

##### 2.3.3.5 Salt Tolerance Test

To assess the Na^+^ requirement, cells were cultured in 1% tryptone broth (Oxoid Ltd., Basingstoke, England) containing 0% and 6% (w/v) NaCl (Merck, Germany). The inoculated media were incubated in a shaking water bath at 37°C for 18–24 h. Turbidity in the broth was used to indicate a positive result (Choopun et al. 2002).

### 2.4 Bacterial Risk Indices

In this study, three bacterial risk indices were selected depending on the microbial limit for fresh fish recommended by the FAO (Sheng and Wang 2021; Butler et al. 2006). Total plate count value less than 10^5^ CFU/g was classified as ‘Good’ quality fish, between 10^6^ and 10^7^ was ‘Acceptable’ limit and more than 10^7^ was indicated as ‘Unacceptable’.

### 2.5 Statistical Analysis

Statistical analyses were done using *R* version 4.4.0 software. Total plate count data were subjected to one‐way ANOVA to observe significant variation. Visualisation of data was done using *R* version 4.4.0 and Adobe Illustrator version 28.0.

## 3 Results

### 3.1 Identification of Pathogenic Bacteria in the Samples

In this study, confirmation of the six pathogenic bacteria was done using the cultural, microscopic and biochemical examination (Table 2). *E. coli* showed a pink colour colony in MacConkey agar and a greenish metallic colour on EMB agar plates. In case of *Salmonella*, the presence of black spots in the centres of SS agar was visual, whereas for *Shigella*, the colonies were colourless. On TCBS agar, colonies of *Vibrio cholerae* showed yellow coloration while *V. parahaemolyticus* and *V. vulnificus* displayed green colonies. The biochemical analysis showed that all the microorganisms tested positive for catalase and methyl red. Members of the Vibrio species tested positive for the oxidase reaction, while *E. coli*, Salmonella spp. and Shigella spp. were negative for the oxidase reaction. During TSI testing, *E. coli* and *V. cholerae* exhibited the acid/acid (A/A) reaction and *Salmonella* spp., *Shigella* spp., *V. parahaemolyticus* and *V. vulnificus* showed alkaline/acid (K/A) state. Again, H_2_S production was found in *Salmonella* spp. and both *E. coli* and *Salmonella* spp. produced gas. To identify *Vibrio* species growth in 0% NaCl and 6% NaCl was observed that *V. cholerae* grew in both saline levels, but *V. parahaemolyticus* and *V. vulnificus* only survived and reproduced in 6% NaCl.

**TABLE 2:** Cultural, morphological and biochemical observation of the pathogenic bacteria isolated from the fish, soil and water samples.

Test	*E. coli*	*Salmonella*	*Shigella*	*V. cholerae*	*V. parahaemolyticus*	*V. vulnificus*
**Agar**	MacConkey Agar: Pink colonies	SS Agar: Colourless with black centre	SS Agar: Colourless colonies	TCBS Agar: Yellow colonies	TCBS Agar: Green colonies	TCBS Agar: Green colonies
	EMB Agar: Metallic green sheen colonies	XLD Agar: Red with black centres	XLD Agar: Red/pink colonies, no black			
**Gram Stain and Microscopic shape**	Gram‐negative and rod‐shaped	Gram‐negative and rod‐shaped	Gram‐negative and rod‐shaped	Gram‐negative and comma‐shaped rods	Gram‐negative and rod‐shaped	Gram‐negative, curved‐shaped rod
**Motility**	Positive	Positive	Negative	Positive	Positive	Positive
**Oxidase**	Negative	Negative	Negative	Positive	Positive	Positive
**Catalase**	Positive	Positive	Positive	Positive	Positive	Positive
**Methyl Red (MR)**	Positive	Positive	Positive	Positive	Positive	Positive
**Triple Sugar Iron (TSI)**	A/A	**K/A**	**K/A**	A/A	**K/A**	K/A
**H**_**2**_ **S**	Negative	Positive	Negative	Negative	Negative	Negative
**Gas Production**	Positive	Positive	Negative	Negative	Negative	Negative
**Growth in 0% NaCl**	N/A	N/A	N/A	Positive	Negative	Negative
**Growth in 6% NaCl**	N/A	N/A	N/A	Positive	Positive	Positive

### 3.2 Bacterial Load and Presence in Different Fish Samples

**FIGURE 2:**
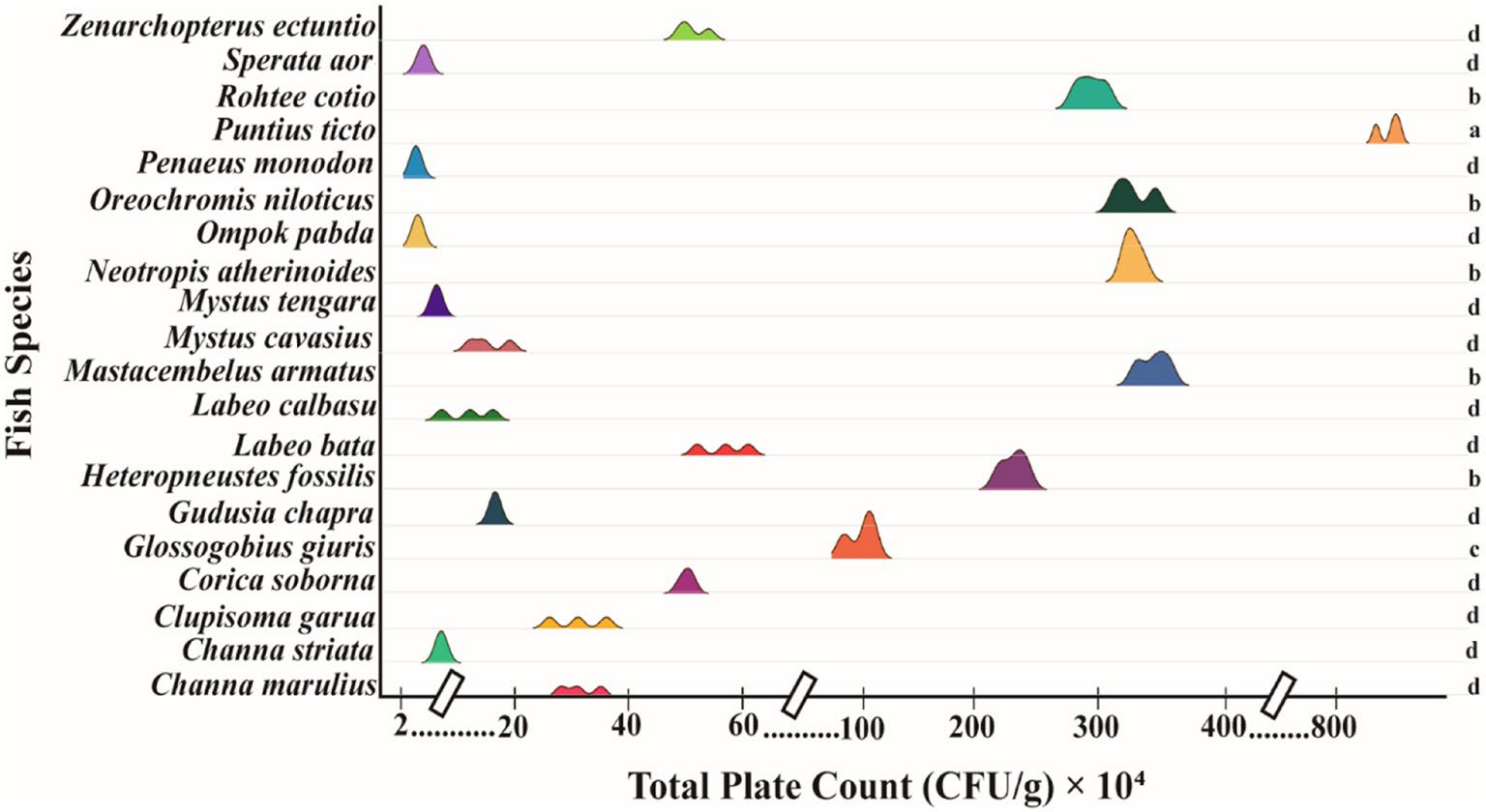
Bacterial load in different fish species.

The bacterial load in 20 fish species varied from 2.45 × 10^4^ ± 2.5 × 10^3^ CFU/g to 8.53 × 10^6^ ± 2.05 × 10^5^ CFU/g. The highest bacterial load was reported in *Puntius ticto* with 8.53 × 10^6^ ± 2.05 × 10^5^ CFU/g, whereas the lowest value was found in *Penaeus monodon* with 2.45 × 10^4^ ± 2.5 × 10^3^ CFU/g (Figure 2). Comparatively lower numbers of bacterial colonies were observed in the samples from *Ompok pabda*, *Sperata aor*, *Mystus tengra*, and *Channa striata*, at 2.77 × 10^4 ± 1.53 × 10^3^, 3.74 × 10^4 ± 6.45 × 10^3^, 6.08 × 10^4 ± 3.55 × 10^3^, and 6.90 × 10^4 ± 3.61 × 10^3^ CFU/g, respectively. The bacterial load in the fish samples showed significant variation across different locations (*p* < 0.05). In the case of 20 fish samples, 13 fish species were categorised as ‘Good’, indicating safe for consumption. The bacterial load in the 13 fish species varied from 2.45 × 10^4^ ± 2.5 × 10^3^ CFU/g to 5.67 × 10^5^ ± 4.51 × 10^4^ CFU/g. The rest of the seven fish species were identified as ‘Acceptable’, the range varying from 1.23 × 10^6^ ± 1.14 × 10^5^ CFU/g to 8.53 × 10^6^ ± 2.05 × 10^5^ CFU/g.

**FIGURE 3:**
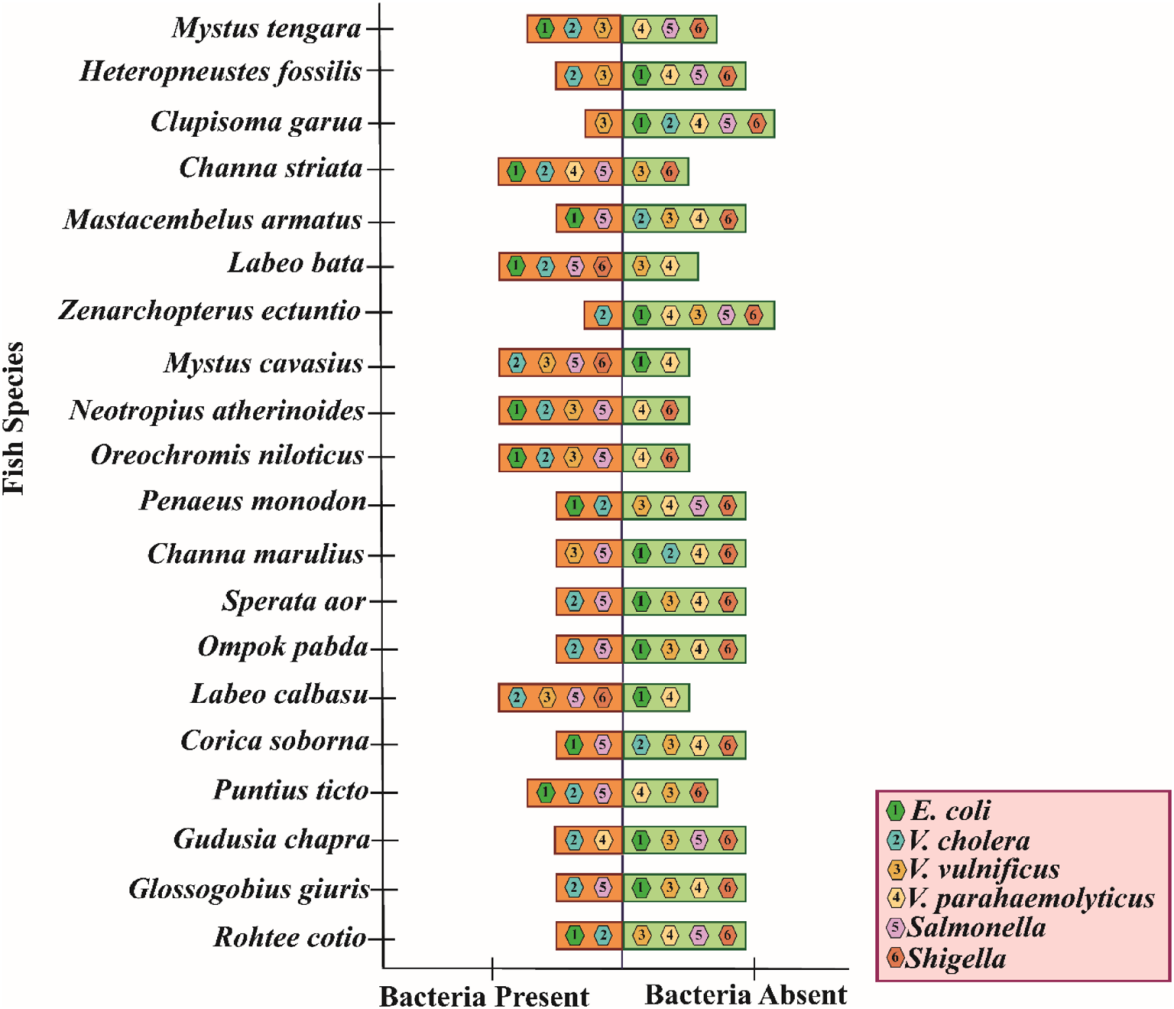
Presence of six pathogenic bacteria in different fish species.

Besides the bacterial load, different kinds of pathogenic bacteria were found present in the 20 different fish samples shown in Figure 3. *E. coli* was present in nine fish species, *V. cholerae* was in 15 species, *V. vulnificus* was in 8 species, *V. parahaemolyticus* was in four species, *Salmonella* in 13 species and *Shigella was* in three species. In *Labeo calbasu, Mystus tengra*, *Labeo bata* and *Channa striata*, four of the five bacterial isolates were present: *Neotropius atherinoides*, *Oreochromis niloticus*, *Mystus tengra*, *Labeo bata*, *Puntius ticto* and *Heteropneustes fossilis*, three species.

### 3.3 Bacterial Load and Presence of Pathogenic Bacteria in Soil Samples

**FIGURE 4:**
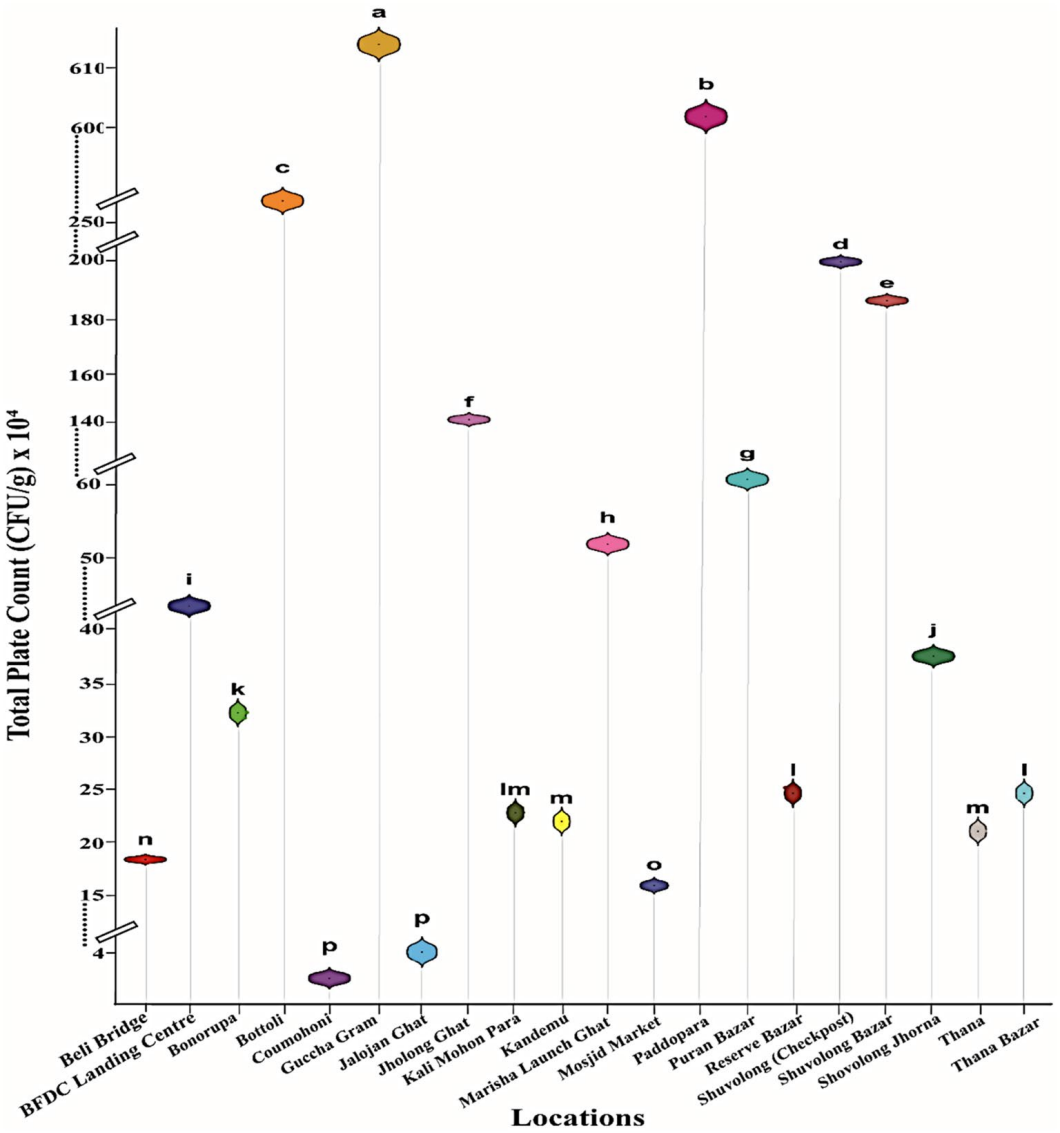
Bacterial load in soil samples collected from the adjacent areas of Kaptai Lake and Baghaichari region.

The bacterial load in soil varied from 3.31 × 10^4^ ± 1.0 × 10^3^ CFU/g to 6.13 × 10^6^ ± 1.0 × 10^4^ CFU/g, with the highest and lowest found in the Baghaichari region, Guccha Gram and Coumohoni, respectively. On the other hand, the bacterial load of soil samples collected from the adjacent areas of the lower region of Kaptai Lake varied from 3.94 × 10^4^ ± 1.4 × 10^4^ CFU/g to 1.98 × 10^6^ ± 1.0 × 10^4^ CFU/g. Soil samples from Shuvolong (Check post) showed higher amounts of bacterial load and lower amounts were recorded from Jalojan Ghat. The bacterial load in the soil samples showed significant variation across different locations (*p* < 0.05) (Figure 4).

**FIGURE 5:**
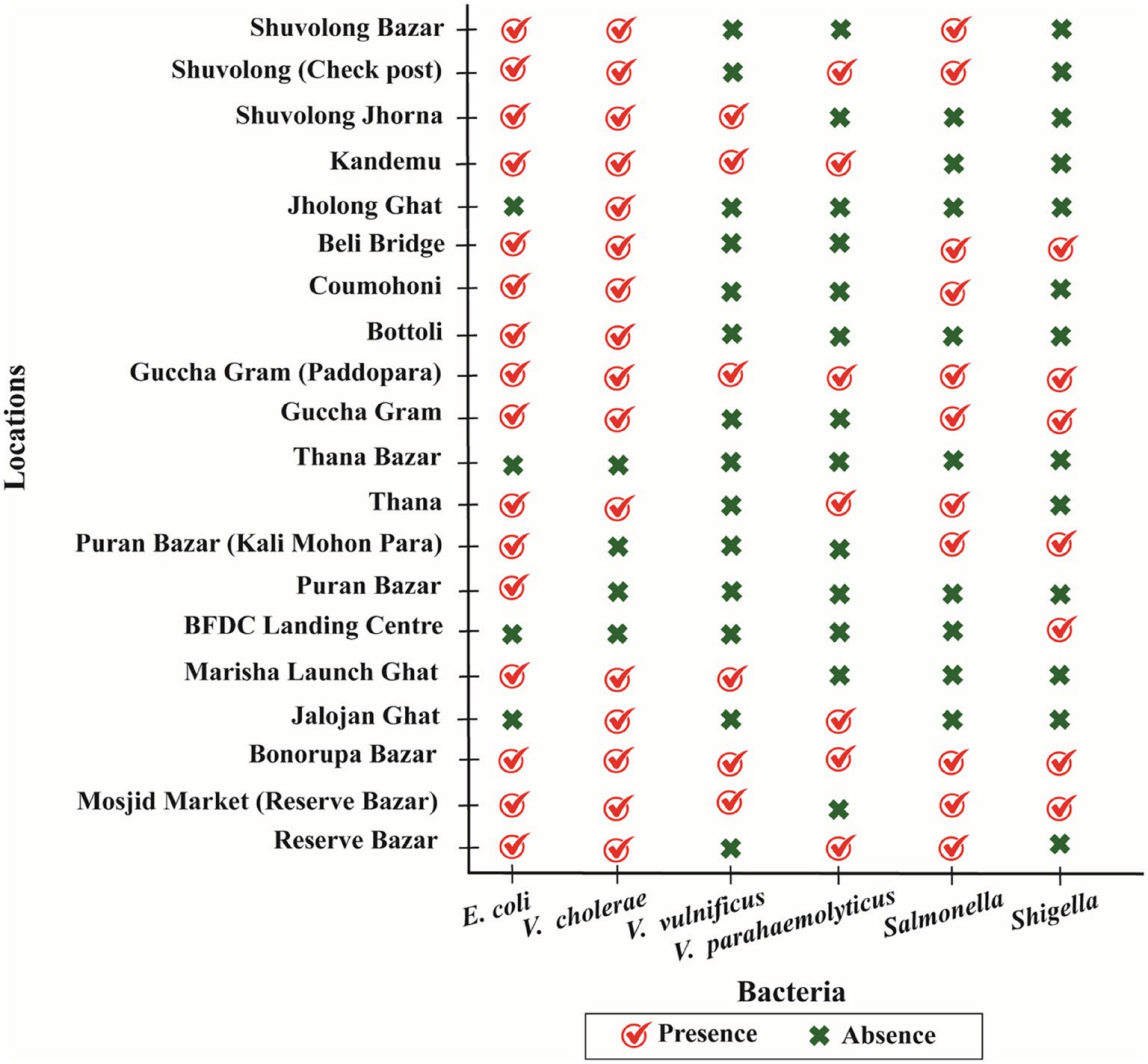
Presence of pathogenic bacteria in soil samples.

Additionally, in the soil samples, the most prevalent bacterial isolates were *E. coli* and *V. cholerae* which were found in 16 locations. Other bacterial isolates like *V. vulnificus* were reported in six locations, *V. parahaemolyticus* and *Shigella* in seven locations and lastly, *Salmonella* in 11 locations (Figure 5).

### 3.4 Bacterial Load and Presence of Pathogenic Bacteria in Different Water Samples

**FIGURE 6:**
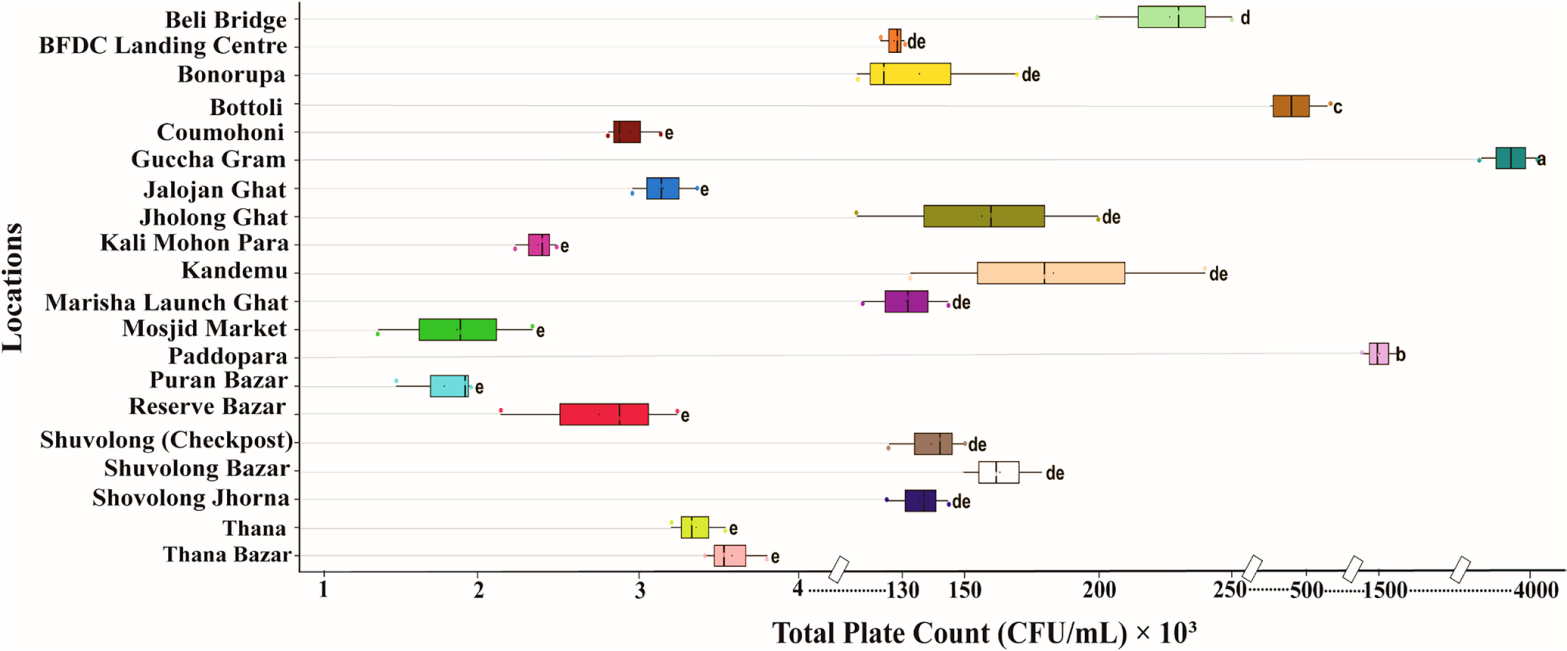
Bacterial load in different water samples.

The bacterial load in water samples collected from 20 locations showed significant variation across different locations (*p* < 0.05), ranging from 1.75 × 10^4 ± 2.61 × 10^3 CFU/mL to 3.90 × 10^6 ± 2.0 × 10^5 CFU/mL. The highest load was recorded in Guccha gram and the lowest in the Puran Bazar, both from the Baghaichari region. On the contrary, in the lower region of Kaptai Lake, the highest load was recorded in Bonorupa with 1.33 × 10^5^ ± 3.21 × 10^4^ CFU/mL and the lowest in the Reserve Bazar, 2.71 × 10^4^ ± 5.61 × 10^3^ CFU/mL. A moderate level of load was observed in Bonorupa, Beli Bridge, Shuvolong Jhorna, Shuvolong Check Post, Shuvolong Bazar with 1.33 × 10^5^ ± 3.21 × 10^4^ CFU/g, 2.27 × 10^5^ ± 2.52 × 10^4^ CFU/g, 1.33 × 10^5^ ± 1.16 × 10^4^ CFU/g, 1.38 × 10^5^ ± 1.43 × 10^4^ CFU/g and 1.63 × 10^5^ ± 1.50 × 10^4^ CFU/g, respectively (Figure 6). The bacterial load in the fish samples.

**FIGURE 7:**
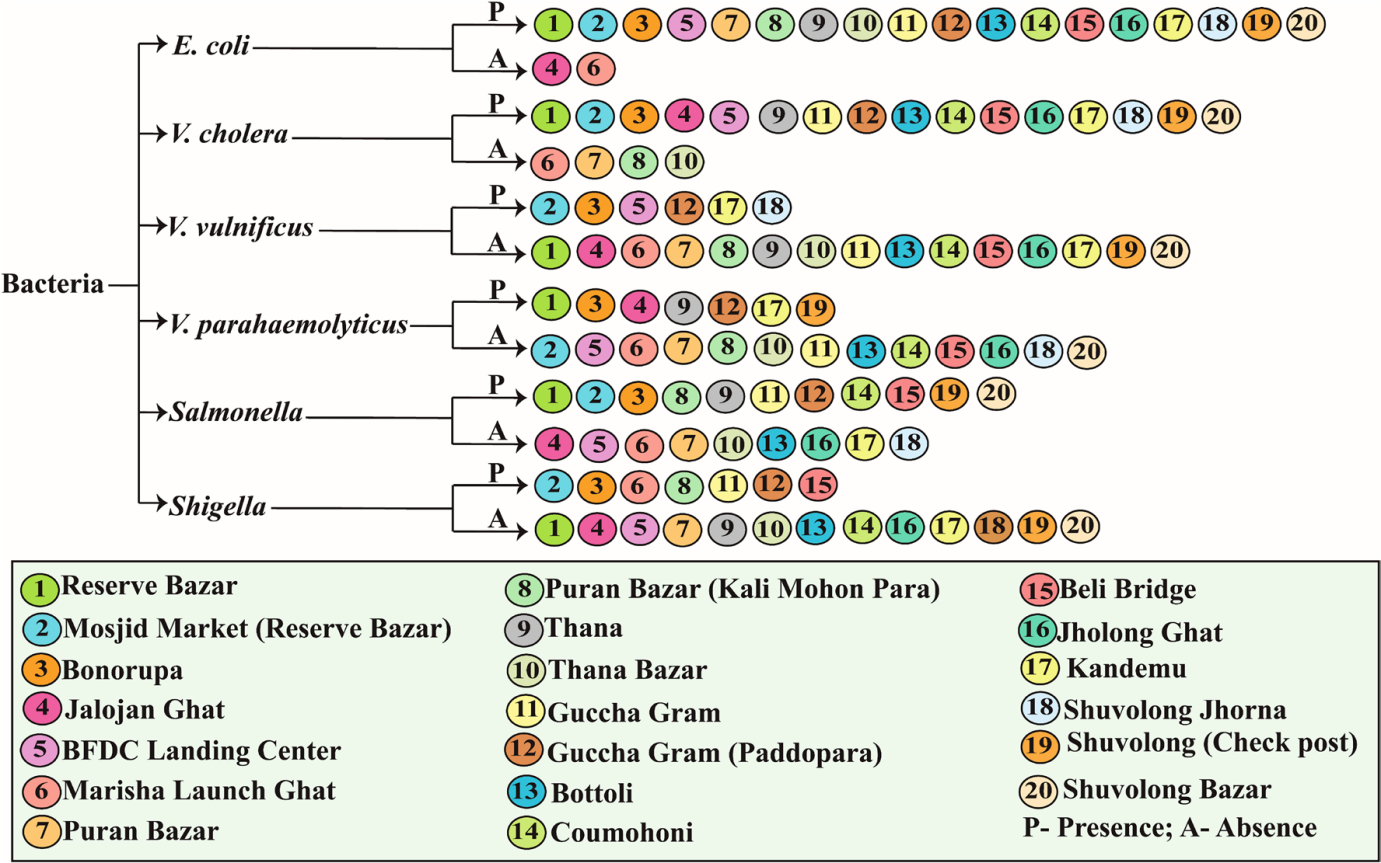
Presence of pathogenic bacteria in water samples collected from the lower region of Kaptai Lake and Baghaichari region.

Bacteria isolated and identified from different water samples are shown in Figure 7. In the water samples, *E. coli*, *Salmonella* and *Shigella* were detected in 15, 11 and 7 locations, respectively, whereas *V. cholerae*, *V. vulnificus*, and *V. parahaemolyticus* were identified in 16, 6 and 7 places, respectively.

### 3.5 Relationship Among Microbial Load of Soil, Water and Fish Species

**FIGURE 8:**
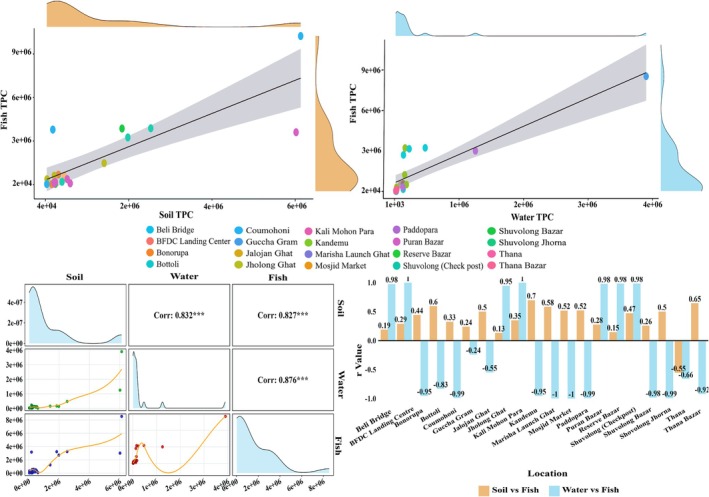
Relationship among the bacterial load in soil, water and fish samples collected from 20 different locations of Kaptai Lake.

There is a positive relation between the microbial load of fish and soil, as well as fish and water samples (*r* > 0.8), meaning that higher levels of bacterial load in soil and water resulted in higher levels of microbial load in fish. *Puntius ticto* resulted in a higher amount of bacterial load with 8.53 × 10^6^ ± 2.05 × 10^5^ CFU/g, which was collected from Guccha gram. Moreover, a higher level of bacterial load in soil and water was also reported in Guccha Gram with values 6.13 × 10^6^ ± 1.0 × 10^4^ CFU/g and 3.90 × 10^6 ± 2.0 × 10^5^ CFU/mL, respectively. A similar trend was also observed in *Rohtee cotio* collected from Poddopara which recorded the 2nd highest number of bacterial loads with 3.00 × 10^6 ± 1.15 × 10^5^ and both soil and water samples also showed the 2nd highest number of loads with 6.02 × 10^6 ± 1.00 × 10^4^ and 1.25 × 10^6 ± 1.31 × 10^5^, respectively. In contrast, in Coumohoni, the correlation among sources remained positive, showing a lower level of bacterial load in soil and water with 3.31 × 10^4 ± 1.00 × 10^3^ and 2.90 × 10^6 ± 1.74 × 10^4^, respectively, which resulted in a lower level of load in *Ompok pabda* (2.77E+04). Again, BFDC Landing Centre exhibited a moderate level of contamination, recording soil TPC at 4.54 × 10^5 ± 5.0 × 10^3^ CFU/g, water TPC at 8.87 × 10^4 ± 5.13 × 10^3^ CFU/mL, and fish TPC at 5.01 × 10^5 ± 1.03 × 10^4^ CFU/g. The high correlation scores in most places (from 0.5 to 1) clearly show that environmental hygiene matters most for the safety of fish (Figure 8).

## 4 Discussion

### 4.1 Bacterial Load in Samples

The findings of the present study revealed considerable variation in bacterial loads across fish, soil, and water samples collected from different locations in and around Kaptai Lake and Baghaichari, reflecting the influence of both environmental and anthropogenic factors. The total plate count (TPC) in fish samples ranged from 2.45 × 10^4^ to 8.53 × 10^6^ CFU/g, with *P. ticto* exhibiting the highest load and *P. monodon* the lowest. According to the microbial quality classification by the International Commission on Microbiological Specifications for Foods (Foods 1974) which considers counts below 5 × 10^5^ CFU/g as “good” and those between 5 × 10^5^ and 10^7^ CFU/g as “acceptable,” 13 of the 20 fish species analysed in this study can be classified as microbiologically safe or “good,” while the remaining seven species were “acceptable.” This distribution of bacterial loads is consistent with earlier studies conducted in Bangladesh and other tropical countries, where TPCs in fish typically fall within the range of 10^4^ to 10^8^ CFU/g, depending on the species, habitat, and post‐harvest handling practices (Amin et al. 2024). The comparatively higher bacterial load in *P. ticto* may be attributed to its benthic feeding behaviour and increased exposure to sediment‐borne bacteria. Similar trends were observed by Olafsdottir et al. (1997), who reported that fish species with bottom‐feeding habits often harbour higher microbial loads due to prolonged contact with contaminated substrates. Moreover, bacterial colonisation is further influenced by water quality, temperature, and fish physiology, all of which play significant roles in determining the microbiota of aquatic organisms (Rathod et al. 2022).

Soil samples from the corresponding sampling sites revealed bacterial loads ranging from 3.31 × 10^4^ to 6.13 × 10^6^ CFU/g. The highest values were recorded in the Baghaichari region, specifically Guccha Gram, while the lowest were found in Coumohoni. These findings mirror those of Babuji et al. (2023), who documented total viable bacterial counts between 10^4^ and 10^7^ CFU/g in agricultural and riverside soils across rural Bangladesh. Soil in such regions often receives continuous input from organic waste, animal faeces, decomposing matter, and irrigation water, all of which contribute to elevated bacterial densities (Rathod et al. 2022). The microbial content of soil also fluctuates based on season, rainfall, and human activities, with dry seasons typically favouring microbial proliferation due to the accumulation of organic matter and reduced leaching (Sarkar et al. 2019).

Water samples from the same locations showed bacterial loads ranging from 1.75 × 10^4^–3.90 × 10^6^ CFU/mL. Similar to the fish and soil data, the highest waterborne bacterial load was found in Guccha Gram, while the lowest was recorded in Puran Bazar. These levels are consistent with the microbial contamination levels of various surface waters in Bangladesh, particularly those receiving domestic waste, sewage, or agricultural runoff. Studies on the Buriganga and Padma Rivers reported TPC levels ranging from 1 × 10^5^–4.2 × 0^6^ CFU/mL, and faecal coliform counts often exceeded acceptable limits (Saha et al. 2009). The seasonal fluctuation of microbial populations in surface water bodies is also well documented, with peak levels typically occurring in the dry season when dilution capacity is reduced and temperature is favourable for bacterial growth (Jahan et al. 2017).

The significant correlation (*p* < 0.05) between elevated microbial loads in fish, soil, and water in specific locations such as Baghaichari suggests a strong environmental influence on fish microbiology. These findings aligned with the concept of environmental microbial transfer, wherein bacteria from soil and water colonise the external and internal surfaces of fish through direct contact or ingestion. Xu et al. (2022) emphasised that microbial contamination in aquaculture is often linked to the quality of surrounding water and sediments, especially in ecosystems lacking proper waste management infrastructure. In the present study, Guccha Gram consistently yielded the highest bacterial loads across all three sample types, reinforcing the notion that this area may be an environmental hotspot of microbial contamination.

**FIGURE 9:**
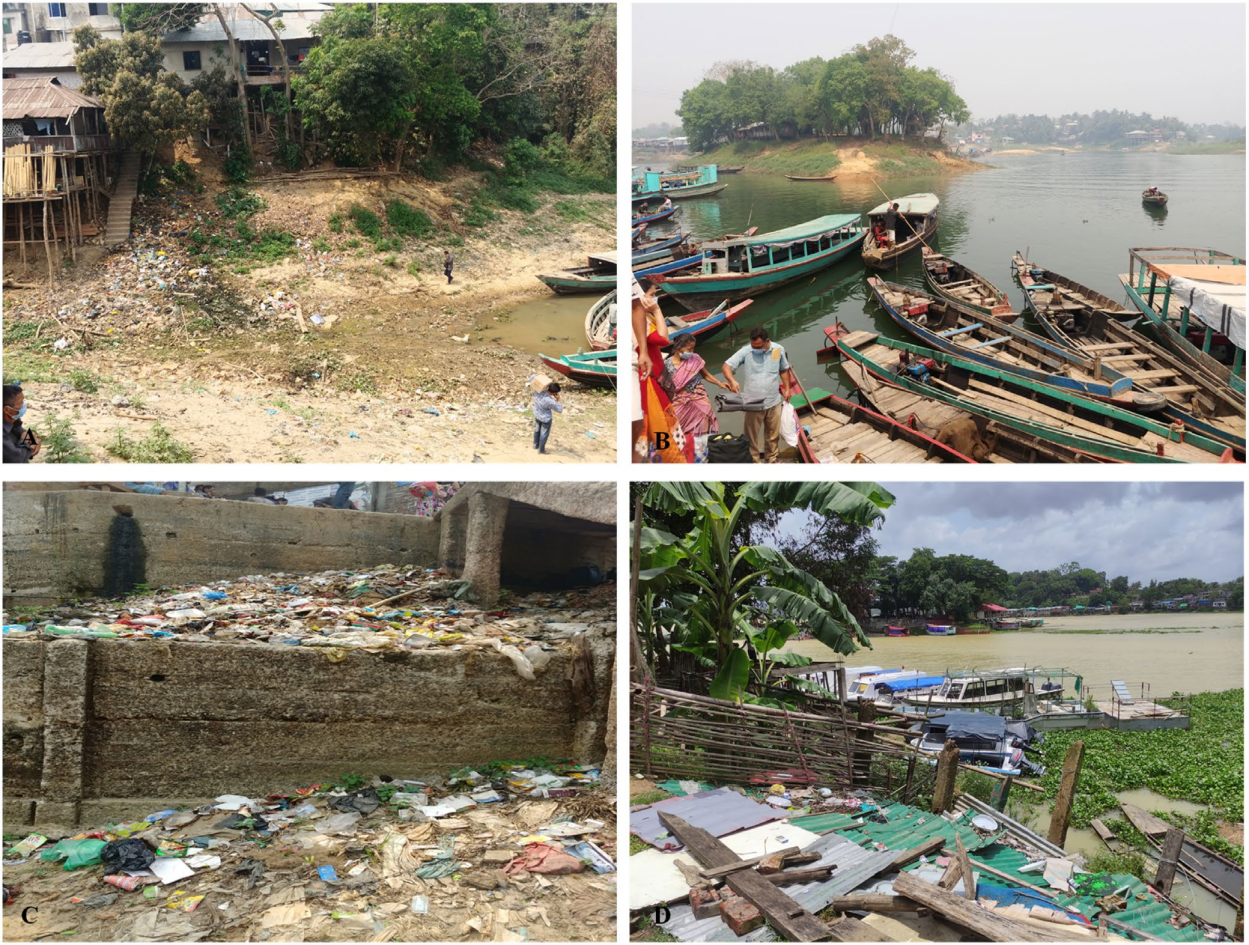
Sources of contamination in the study area (A) Household wastes polluting lake water; (B) Busy boat terminal; (C) Dustbin near the lake; (D) Lakeside littered with debris.

Additionally, the data obtained in this study suggest that microbial contamination is not uniformly distributed, but rather site‐specific and influenced by localised conditions. This observation is supported by Kabir et al. (2023), who found that bacterial pollution in the Buriganga River varied considerably between urban and peri‐urban areas due to differences in domestic and industrial waste discharge. Similarly, environmental surveys in Cameroon showed that ponds with poor water exchange and high nutrient loads had significantly higher bacterial densities in sediment, water, and fish (Tsafack et al. 2021), reinforcing the universality of these findings (Figure 9).

Although the current study focused on total bacterial counts, it is important to note that high TPC may indicate increased spoilage potential but not necessarily the presence of pathogenic organisms. However, multiple studies have reported the presence of *Escherichia coli*, *Salmonella*, and *Vibrio* spp. in fish and water samples from Bangladeshi markets and rivers, especially when total bacterial loads exceed 10^6^ CFU/g or ml (Kabir et al. 2023). The findings of this study are consistent with regional and global literature and emphasise the importance of understanding environmental contributions to foodborne microbial risks. Further research should explore pathogen‐specific profiles and antibiotic resistance patterns to provide a more detailed risk assessment framework.

### 4.2 Pathogenic Bacteria in Fish, Soil and Water Samples

In Kaptai Lake, pathogenic bacteria such as *E. coli*, *Vibrio cholerae*, and *Salmonella* sp. were found to be prevalent in fish, shellfish, soil, and water samples, which were primarily attributed to contamination from animal faeces (Saif et al. 2017). While the microbiological quality of the fish falls within acceptable ranges according to published guidelines, concerns arise due to the potential for infections, especially given the compromised immune systems of many individuals within communities (Abdelhamid et al. 2006; Sheng and Wang 2021).

Different studies examined the isolation and identification of pathogenic bacteria in various fish species from different water bodies, where common pathogens isolated included *E. coli*, *Staphylococcus aureus*, *Salmonella typhi*, *Pseudomonas aeruginosa*, and *Aeromonas species* (Sharma et al. 2013; Sichewo et al. 2013; Akila 2018; Abedin et al. 2020). These bacteria were found in fish tissues such as gills, intestines, skin, and liver. In the present study, *E. coli*. was present in the highest number of fish species and the presence of enteric bacteria in fish was identified as an indicator of faecal contamination and water pollution (Sichewo et al. 2013; Akila 2018). The bacterial load in fish tissues often exceeded recommended public health standards recommended by FAO (> 10^7^ CFU/g) (Sichewo et al. 2013; Butler et al. 2006). Furthermore, the presence of *Vibrio vulnificus* in samples from certain stations raises alarm due to the potential for severe infections, particularly among vulnerable populations (Abdelhamid et al. 2006). The reasons behind the high microbial load of the fish sample might be pollution, agricultural runoff, and the activity of local people in the lake. These findings highlight the potential health risks associated with improperly handled or prepared fish and emphasise the importance of monitoring water quality and fish health in aquaculture and natural water bodies.

Few researchers collectively demonstrate the prevalence of pathogenic bacteria in various water bodies and soil samples. Common isolates include *E. coli, Salmonella* sp., *Staphylococcus* spp., *P. aeruginosa*, *and Vibrio* sp., among others (Ulfat et al. 2022; Parida et al. 2012; Sichewo et al. 2013). These bacteria pose significant health risks, causing diseases such as gastroenteritis, cholera, and urinary tract infections (Ulfat et al. 2022). The research highlights the unsuitability of contaminated water for domestic use, irrigation, or recreational activities without proper treatment (Ulfat et al. 2022; Sichewo et al. 2013). (Ava et al. 2020) discovered that the incidence of *Salmonella* was higher in scum samples (93.8%), while for *E. coli*, the higher contamination estimates were in open water samples (81.3%). Additionally, water and fish samples also tested positive for *Salmonella*, with percentages of 87.5% and 57.8%, respectively. A similar trend was observed by (Faridullah et al. 2016), who found *Salmonella* positive samples in water, scum, and shrimp at rates of 43.7%, 62.5%, and 20% respectively. Their study revealed contamination of water, scum, and fish with *Salmonella* and *E. coli*, with percentages of positive *Salmonella* and *E. coli* isolates at 31.03% and 50.00% respectively (Faridullah et al. 2022). A pathogenic *Vibrio* that has infected the aquatic system joins the shellfish microflora. When ingested by fish, the dangerous pathogenic *Vibrio* can result in potentially fatal foodborne infections and pose a serious risk to public health. The studies emphasise the need for improved water management, treatment processes, and public awareness to mitigate the risks associated with pathogenic bacteria in water bodies and soil (Ulfat et al. 2022).

Of particular concern is the high prevalence of *Salmonella* sp. in fish samples, as well as its presence in soil and water samples, highlighting the significant health risk associated with contaminated water sources. The detection of other enteric bacteria further underscores the need for strict regulations, monitoring procedures, and food safety education initiatives targeting suppliers and consumers (Sadeghi et al. 2007; Cabral 2010). Potential human sources include wastewater discharge, sewage leaks, and failing septic tanks and drain fields. Additionally, partially treated sewage is sometimes directly discharged into rivers, intentionally or unintentionally, often exacerbated by storm events, which have been shown to increase *E. coli* counts in water significantly (Cho et al. 2020). These findings underscore the importance of comprehensive water quality assessment and proactive measures to prevent waterborne outbreaks and associated health risks. Indicators of contamination, such as the presence of coliform bacteria, highlight the extent of environmental pollution in Kaptai Lake. Elevated levels of thermotolerant coliform species further indicate the risks associated with water consumption, particularly for susceptible individuals (Faridullah et al. 2022).

However, our findings suggested that Kaptai Lake is contaminated with pathogenic bacteria, rendering it unsuitable for drinking and household use. Urgent action is needed to address sources of contamination, improve water quality, and protect public health in affected communities. Further research is warranted to better understand the types and frequency of diseases among residents in the vicinity of Kaptai Lake, informing targeted interventions and mitigation strategies.

## 5 Conclusion

This investigation underscores the urgent need to monitor bacterial presence in fish, water, and soil samples to uphold public health standards. The identification of pathogenic strains such as *E. coli*, *Vibrio cholerae*, *Vibrio vulnificus*, and *Salmonella* sp. in Kaptai Lake highlights a significant risk of intestinal infectious diseases if left unchecked. The presence of such bacteria can cause bacterial diseases in fish, as well as cause zoonotic diseases in humans when consumed. Proper management, like a proper waste disposal system and proper sanitary facilities for the households, should be built so that such wastes do not accumulate in the lake water. Again, the microbial loads exceed the safety thresholds set by the National Agency for Food, Drug Administration, and Control (NAFDAC), emphasising the necessity for strict adherence to proper harvesting, handling, and cooking practices to mitigate foodborne illnesses. Implementation of a buffer zone to reduce agricultural runoff, regular monitoring of the key microbial indicators, may result in a reduced level of microbial contamination in the lake. Thus, this research provides essential insights into the microbial landscape of Kaptai Lake and underscores the importance of proactive measures to safeguard against potential outbreaks of foodborne diseases.

## Author Contributions

**Susmita Chakma:** conceptualization, visualization, data curation, writing – original draft, writing – review and editing. **Hrishika Barua:** conceptualization, visualization, data analysis, data curation, writing – review and editing. **Aklima Akter:** data curation, writing – review and editing. **Shama Afroze:** data curation, editing and draft correction. **Md Faisal:** investigation, funding acquisition, visualization, writing – review and editing, project administration, supervision. **Nurul Absar Khan:** monitoring and supervision.

## Funding

This work was supported by Chattogram Veterinary and Animal Sciences University, MS Research Grant.

## Ethics Statement

This study did not need any ethical approval because all the fish samples were collected dead in the local markets where they are sold to people to be consumed. There were no animals that were used or euthanised in the research.

## Consent

The authors have nothing to report.

## Conflicts of Interest

The authors declare no conflicts of interest.

## Data Availability

Data available on request from the authors.
