# Correction to ‘Isolation and Identification of Pathogenic Bacteria Present in Commercially Important Fish, Shellfish, Water, and Soil Samples of Kaptai Lake, Bangladesh’

**DOI:** 10.1111/1758-2229.70367

**Published:** 2026-05-31

Chakma, S. , H. Barua , A. Akter , S. Afroze , M. Faisal , and M. N. A. Khan . 2025. “Isolation and Identification of Pathogenic Bacteria Present in Commercially Important Fish, Shellfish, Water, and Soil Samples of Kaptai Lake, Bangladesh.” Environmental Microbiology Reports 17, no. 6: e70252. 10.1111/1758-2229.70252 41339126 PMC12674966

The sixth author's name is incorrectly listed in the byline. It currently reads ‘Nurul Absar Khan’, but it should be corrected to ‘Mohammed Nurul Absar Khan’. Mohammed should be added to the given name.

The online version of the article has been corrected.

We apologize for this error.

